# Dialdehyde Cellulose Nanocrystals and Proanthocyanidins Reinforced Soy Protein Isolate Films for Blueberry Preservation

**DOI:** 10.3390/polym17212821

**Published:** 2025-10-23

**Authors:** Jiapeng Wei, Kehao Fan, Manting Meng, Zhiyong Qin, Ningjing Sun

**Affiliations:** 1 College of Resources and Environment Sciences, Baoshan University, Baoshan 678000, China; plianasp@163.com; 2 State Key Laboratory of Featured Metal Materials and Life-cycle Safety for Composite Structures, School of Resources, Environment and Materials, Guangxi University, Nanning 530004, China; fankehaoo@163.com (K.F.);

**Keywords:** soy protein isolate, dialdehyde cellulose nanocrystals, schiff base crosslinking, proanthocyanidins, active food packaging, synergistic reinforcement, covalent cross-linking, antimicrobial films, blueberry preservation

## Abstract

Exhibiting significant potential for sustainable packaging due to their renewability and biodegradability, soy protein isolate (SPI) films are nevertheless critically hampered by inherent brittleness, poor water resistance, and a lack of bioactivity. Herein, we demonstrate a hierarchical multi-network strategy that transforms SPI into a high-performance, functional biocomposite. A robust covalent backbone was forged via Schiff base cross-linking between SPI and dialdehyde cellulose nanocrystals (DACNCs) derived from agricultural biomass, while proanthocyanidins (PAs) were strategically incorporated to create a secondary, pervasive hydrogen-bonding network. This hierarchical architecture effectively overcomes the typical trade-offs between mechanical strength and functionality seen in singly modified biopolymers, unlocking a suite of remarkable performance enhancements. The optimized film exhibited a 491% increase in tensile strength (to 15.54 MPa) and elevated thermal stability to 330 °C. Critically, the film was endowed with potent functionalities, including complete UV-blocking, high antioxidant capacity (93.2% ABTS scavenging), and strong, broad-spectrum antimicrobial activity. The film’s practical efficacy was validated in a preservation test, where the coating extended blueberry shelf life by inhibiting fungal spoilage and reducing weight loss by nearly 30% relative to uncoated controls after 15 days of storage. This work provides a powerful framework for developing advanced biocomposites with tailored properties for active food packaging and beyond.

## 1. Introduction

In the global response to plastic pollution, the development of fully biodegradable packaging materials from renewable biomass has become a central focus of both scientific and industrial research [1]. Among the diverse array of bio-based polymers, soy protein isolate (SPI) exhibits significant potential, valued for its abundance, low cost, complete biodegradability, and excellent film-forming properties [2]. Notably, under dry conditions, the gas barrier performance of SPI films can even surpass that of many polysaccharide-based films, presenting a unique advantage for specific food packaging applications [3,4].

Despite this potential, the commercialization of neat SPI films is confronted by two major challenges: first, the protein network, maintained primarily by non-covalent interactions, results in materials with insufficient mechanical strength and high brittleness; second, the abundance of polar groups on its molecular chains imparts strong hydrophilicity, leading to extremely poor water resistance [4].

Therefore, researchers have been dedicated to enhancing its overall properties through various modification strategies. Common approaches can be broadly categorized into physical modification, chemical modification, and the incorporation of active agents. Physical modification often involves blending SPI with other biopolymers like starch or chitosan to achieve synergistic properties, or incorporating nano/micro-scale fillers such as nanoclays, zinc oxide nanoparticles, or unmodified cellulose nanocrystals for physical reinforcement. Chemical modification aims to create more stable networks, typically through covalent cross-linking using agents like aldehydes and epoxides, or by grafting new functional groups onto the protein backbone to alter properties such as hydrophilicity [5]. More recently, the focus has shifted towards developing ‘active packaging’ by incorporating functional components (e.g., plant polyphenols, essential oils) to impart antioxidant and antimicrobial activities. Among these, a key strategy of this research focuses on a combination of chemical cross-linking and the incorporation of active functional components to develop high-performance active packaging [3,6].

To this end, a direct strategy is to introduce high-strength nanofillers like cellulose nanocrystals (CNCs). Derived from nature’s most abundant biomass, the inherent rigid rod-like crystal structure and high specific surface area of CNCs enable them to physically enhance the mechanical and barrier properties of the protein matrix by inducing protein conformational rearrangement and promoting a more homogeneous and compact network through enhanced hydrogen bonding [7]. However, the interaction between CNCs and the SPI matrix is primarily governed by weak non-covalent bonds such as hydrogen bonds, and this tenuous interfacial adhesion limits further improvements in reinforcement, especially in humid environments [7].

Among various modification methods, chemical cross-linking has been demonstrated as one of the most fundamental routes to reinforcing protein networks and enhancing their overall performance [8]. A core strategy of this research involves the utilization of dialdehyde cellulose nanocrystals (DACNCs), prepared via selective oxidation of cellulose from an agricultural byproduct, mulberry branches. The fundamental purpose of introducing DACNCs is to leverage their surface aldehyde groups as covalent anchors for an efficient Schiff base reaction with the amino groups of SPI, thereby transforming discrete nanocrystals into structural nodes within the network [9]. Recent studies have confirmed that utilizing dialdehyde starch or dialdehyde cellulose for Schiff base cross-linking with SPI is a state-of-the-art technique for constructing high-strength, high-stability composite films [10,11]. This strategy is designed to upgrade traditional physical filling to chemical covalent cross-linking, aiming to construct a stable and continuous three-dimensional network backbone within the SPI matrix and, consequently, to resolve the intrinsic problems of a loose structure and water instability at a molecular level.

Subsequent to establishing a stable structural platform, we further endeavor to impart the material with the bio-functionalities essential for active packaging. A substantial body of current research focuses on incorporating various plant extracts, such as essential oils or anthocyanins, into the SPI matrix to confer antimicrobial and antioxidant activities [12]. However, the weak bonding of these small-molecule active agents to the matrix can lead to migration issues. In this context, this study specifically selects proanthocyanidins (PAs), a type of polymeric polyphenol. The novelty of this choice lies in two key aspects: first, the potent bioactivity of PAs as effective natural antioxidants and broad-spectrum antimicrobial agents is well-documented [13,14]. Second, and more critically, the unique polymeric structure of PAs enables them to function as multi-arm molecular glue. Unlike monomeric phenols, a single PA molecule can extend multiple phenolic hydroxyl arms to form an extensive, multi-point hydrogen-bonding network with both the SPI chains and DACNC surfaces [8]. This distinctive non-covalent interaction is anticipated to play a dual role of filling and reinforcing within the covalent framework established by DACNC, fostering a denser and more synergistic composite network. This “dual cross-linking” strategy, which combines a covalent cross-linker (like dialdehyde cellulose) with a plant polyphenol (like tannins), has been demonstrated to synergistically enhance the overall performance of the material [11].

The design rationale of this study is highly aligned with an advancement in the field, namely achieving significant enhancements in material performance through the construction of multiple network systems. This leads to a core scientific question: within the SPI matrix, when a robust covalent network mediated by DACNC and a dense non-covalent network mediated by PA coexist, the interaction mechanism between these two forces of disparate nature and scale remains unclear. Therefore, elucidating whether this interaction yields a synergistic enhancement effect that transcends the simple summation of the individual components is of significant theoretical importance for guiding the design of high-performance biocomposite materials.

Based on the foregoing analysis, this study proposes a central scientific hypothesis: The construction of a hierarchical cross-linked system, composed of a dominant covalent backbone from Schiff base bonds and a pervasive filling network of multi-point hydrogen bonds, can achieve the simultaneous maximization of both the structural stability and the biological functionality of the SPI composite film. We postulate that the DACNC covalent network acts as the steel reinforcement of the architecture, providing macroscopic strength and stability, while the PA hydrogen-bonding network serves as the flexible cement, filling voids and enhancing toughness. Their synergy will create a film with an integrated profile of high strength, excellent water resistance, and potent antioxidant and antimicrobial activities, with overall performance far exceeding what can be achieved by either modification strategy alone. To validate this hypothesis, this work will systematically fabricate and characterize a series of SPI/DACNC/PA composite films to thoroughly elucidate the structure property relationships. The practical efficacy of the optimized film as a high-performance active packaging material will ultimately be evaluated through a food preservation application. This study aims to rigorously demonstrate that all experimental data sets—structural, mechanical, thermal, water-responsive, and biofunctional—are coherently and quantitatively linked by the proposed hierarchical multi-network strategy. We present a comprehensive mechanism that shows how the DACNC covalent backbone imparts essential rigidity and thermal stability, while the PA hydrogen-bonding filling network successfully drives matrix densification, which subsequently controls the stress dispersion, water barrier performance, and functional bioactivity.

## 2. Materials and Methods

### 2.1. Materials

Soy protein isolate (SPI, protein content > 90%), sodium periodate (NaIO_4_), sodium hydroxide (NaOH), proanthocyanidins (PA), 2,2′-diphenyl-1-picrylhydrazyl (DPPH), 2,2′-azino-bis(3-ethylbenzothiazoline-6-sulfonic acid) diammonium salt (ABTS), Mueller-Hinton (MH) agar, nutrient broth, and magnesium nitrate were purchased from Macklin Biochemical Co., Ltd. (Shanghai, China). Mulberry branches (*Morus alba* L.) were obtained from Qianwengtang Co., Ltd. (Bozhou, China). Ethylene glycol was supplied by Tianjin Kemmao Chemical Reagent Co., Ltd. (Tianjin, China). Anhydrous ethanol, concentrated sulfuric acid, sodium chlorite (NaClO_2_), glycerol, and potassium persulfate (K_2_S_2_O_8_) were procured from Kelong Chemical Co., Ltd. (Chengdu, China). Phosphate-buffered saline (PBS, powder) was from Lanjie Technology Co., Ltd. (Beijing, China). *Staphylococcus aureus* (ATCC 25923) and *Escherichia coli* (ATCC 25922) strains were obtained from Guangdong Huankai Microbial Technology Co., Ltd. (Guangzhou, China). All reagents were of analytical grade.

### 2.2. Preparation of DACNC

CNC were prepared from mulberry branches. Briefly, mulberry branch powder was delignified and bleached using a 7.5% (*w*/*v*) NaClO_2_ solution (pH = 3.8) at 90 °C for 3 h. Hemicellulose was subsequently removed by stirring in a 10% (*w*/*v*) NaOH solution for 12 h at room temperature. The resulting purified mulberry cellulose fiber (MCF) was hydrolyzed in 64% (*w*/*w*) H_2_SO_4_ at 45 °C for 90 min. The suspension was then centrifuged (8000 rpm, 5 min) and dialyzed against deionized water for 3 days to yield a CNC suspension.

For oxidation, the pH of the CNC suspension was adjusted to 3. NaIO_4_ was added at a mass ratio of 3.6:1 (NaIO_4_:CNC), and the reaction proceeded at 40 °C for 4 h in the dark. The reaction was quenched with ethylene glycol. The final product was dialyzed for 3 days to obtain the DACNC suspension, which was stored at 4 °C. The aldehyde content was determined to be 1.2 mmol/g using the hydroxylamine hydrochloride method [15].

### 2.3. Fabrication of Composite Films

**Figure 1:**
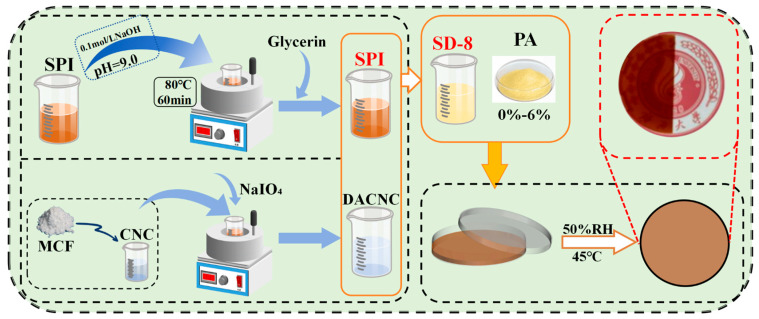
Schematic of the fabrication process for the multi-network SPI/DACNC/PA (SDP) composite films via solution casting.

The overall fabrication process for the multi-network SPI/DACNC/PA (SDP) composite films is illustrated in Figure 1. Composite films were fabricated via a solution casting method. A 2% (*w*/*v*) SPI film-forming solution was prepared by dissolving SPI powder in deionized water, adjusting the pH to 9.0 with 0.1 M NaOH, and heating at 80 °C for 60 min. Glycerol (30 wt% of SPI) was added as a plasticizer.

For SPI/DACNC/PA (SDP) films, DACNC (fixed at 8 wt% of SPI) was first added to the SPI solution and stirred for 2 h. Subsequently, PA was added at various concentrations (0, 1, 2, 3, 4, 5, and 6 wt% of SPI) and stirred for 8 h in the dark. The solutions were degassed via ultrasonication for 10 min. Finally, 30 g of each solution was cast into a Petri dish and dried in a constant climate chamber (KMF115, BINDER, Tuttlingen, Germany) at 45 °C and 50% relative humidity (RH) for 24 h. The designations for the prepared films are listed in Table 1. The dried films were carefully peeled from the Petri dishes by hand. The use of glycerol as a plasticizer ensures sufficient flexibility to allow for easy removal without tearing or damage.

### 2.4. Characterization and Performance Evaluation

#### 2.4.1. Structural and Morphological Analysis

Fourier Transform Infrared Spectroscopy (FT-IR): The chemical structures of the composite films were analyzed using a Fourier transform infrared spectrometer (IRTracer-100, Shimadzu, Kyoto, Japan). For the composite films, spectra were recorded directly using an attenuated total reflectance (ATR) accessory. All spectra were collected over a wavenumber range of 4000–400 cm^−1^ with a resolution of 4 cm^−1^ by accumulating 32 scans.

X-Ray Diffraction (XRD): The crystalline structures of the samples were determined using an X-ray diffractometer (D/MAX 2500 V, Rigaku, Tokyo, Japan). The instrument was equipped with a Cu Kα radiation source (λ = 1.542 Å) and operated at a voltage of 40 kV and a current of 40 mA. The samples were scanned over a 2θ range of 5–45° at a scanning speed of 5°/min.

Scanning Electron Microscopy (SEM): The surface and cross-sectional morphologies of the samples were observed using a scanning electron microscope (Sigma 300, ZEISS, Oberkochen, Germany). The composite films were mounted on an aluminum stub using double-sided carbon tape and sputter-coated with a thin layer of gold for 60 s to enhance conductivity. The images were acquired at an accelerating voltage of 15.0 kV.

Thermogravimetric Analysis (TGA): The thermal stability of the samples was evaluated using a thermogravimetric analyzer (Discovery TGA 550, TA Instruments, New Castle, DE, USA). Approximately 5 mg of each sample was heated from 30 to 600 °C at a constant heating rate of 10 °C/min. The analysis was conducted under a continuous nitrogen atmosphere with a flow rate of 50 mL/min to prevent thermo-oxidative degradation.

X-ray Photoelectron Spectroscopy (XPS): The surface elemental composition and chemical states of the films were analyzed using an X-ray photoelectron spectrometer (Thermo Scientific K-Alpha, Waltham, MA, USA). The instrument was equipped with a monochromatic Al Kα X-ray source (hv = 1486.6 eV) operating at 12 kV and 6 mA. The analysis chamber was maintained at a vacuum of less than 5.0 × 10^−7^ mBar. Survey scans were performed with a pass energy of 150 eV and a step size of 1 eV. High-resolution scans for specific elements (C1s, N1s, O1s) were conducted with a pass energy of 50 eV and a step size of 0.1 eV to obtain detailed chemical state information.

#### 2.4.2. Mechanical and Barrier Properties

Film thickness was measured using a digital micrometer, with three replicates per sample. Tensile strength (TS) and elongation at break (EAB) were determined using a universal testing machine (ZQ-990, Zhiqu Precision Instrument Co., Ltd., Dongguan, China). Film strips (50 mm × 10 mm) were tested at a crosshead speed of 20 mm/min with an initial gauge length of 16.8 mm under controlled conditions (25 ± 1 °C, 50% RH).

The water vapor permeability (WVP) of the films was determined using a gravimetric cup method [16] based on ASTM E96/E96M. Briefly, 3 g of anhydrous calcium chloride was placed in a weighing bottle (permeation area: 1.13 × 10^−3^ m^2^) and sealed with the test film. The sealed bottle was placed in a desiccator containing a saturated sodium chloride solution, which maintained the external environment at 25 °C and 75% relative humidity for 24 h. The WVP was calculated according to Equation (1).

Permeability = (*W* × *d*)/(*A* × *T* × Δ*P*) (1)

where *W* is the weight change in the bottle (kg), *d* is the film thickness (m), *A* is the effective permeation area (m^2^), *T* is the testing time (s), and Δ*P* is the partial pressure difference in water vapor across the film (2376 Pa at 25 °C). All measurements were performed in triplicate.

#### 2.4.3. Water Interaction and Barrier Properties

The ***Swelling Ratio (SR)*** of the films was determined by immersing pre-dried rectangular samples (1 × 2 cm^2^) in 10 mL of deionized water at room temperature for 24 h. After incubation, the samples were gently blotted with filter paper to remove surface moisture. The initial dry weight (*W*_1_) and the swollen weight (*W*_2_) were recorded. *SR* was calculated using Equation (2).

*SR* (%) = (*W*_2_ − *W*_1_)/*W*_1_ × 100% (2)

The ***Water Solubility (WS)*** of the films was determined according to the method reported by Qian [17] et al. Film samples were first dried at 105 °C, and the initial dry weight was recorded as *W*_3_. The samples were then immersed in deionized water and shaken at 25 °C for 24 h. After incubation, the remaining undissolved portions were collected and re-dried at 105 °C to a constant weight (*W*_4_). *WS* was calculated using Equation (3).

*WS* (%) = (*W*_3_ − *W*_4_)/*W*_3_ × 100% (3)

The ***Moisture Content (MC)*** was determined by cutting equilibrated film samples into rectangular strips (1 × 2 cm^2^) and drying them in an oven at 105 °C until constant weight was achieved. The initial weight was recorded as *W*_5_, and the final dry weight as *W*_6_. *MC* was calculated using Equation (4).

*MC* (%) = (*W*_5_ − *W*_6_)/*W*_5_ × 100% (4)

#### 2.4.4. Antioxidant Activity

The antioxidant activity of the composite films was evaluated by measuring their radical scavenging capacity against DPPH and ABTS radicals [18]. For the DPPH assay, 1 mL of film extract was mixed with 4 mL of 0.1 mM DPPH solution in absolute ethanol. The mixture was incubated in the dark at room temperature for 20 min. Absorbance was measured at 517 nm using a UV-Vi spectrophotometer, and the DPPH scavenging rate was calculated using Equation (5).

DPPH (%) = (Dcontrol − Dsample)/Dcontrol × 100% (5)

In the ABTS assay, 7.4 mM ABTS solution in ethanol was mixed with 2.6 mM potassium persulfate solution (1:1, *v*/*v*) and incubated in the dark for 12 h to generate ABTS radicals. The resulting solution was diluted with ethanol until the absorbance at 734 nm reached 0.70 ± 0.02. Then, 1 mL of film extract was mixed with 4 mL of the ABTS working solution and incubated in the dark for 30 min. Absorbance was recorded at 734 nm, and the ABTS scavenging rate was calculated using Equation (6).

ABTS (%) = (Acontrol − Asample)/Acontrol × 100%(6)

#### 2.4.5. Antimicrobial Activity

The antimicrobial activity of the composite films against S. aureus and E. coli was evaluated using a plate counting method. First, film extracts were prepared by soaking 0.1 g of each film sample in 10 mL of sterile phosphate-buffered saline (PBS) and shaking for 24 h. Bacterial suspensions were prepared and diluted to a concentration of approximately 105–106 CFU/mL. Then, 100 µL of the bacterial suspension was mixed with 900 µL of the film solution and incubated at 37 °C for 4 h. The mixture was then serially diluted, and 100 µL of the appropriate dilution was spread onto Mueller-Hinton (MH) agar plates. After incubation at 37 °C for 24 h, the number of colonies was counted. A control group was prepared using pure PBS instead of the film extract. The bacterial reduction rate was calculated using the following equation:

Bacterial reduction (%) = (Ncontrol − Nsample)/Ncontrol × 100%(7)

where, Ncontrol and Nsample are the number of colonies in the control and sample groups, respectively.

### 2.5. Blueberry Preservation Application

Fresh blueberries were coated by immersion in different film-forming solutions (CK, SD-8, SDP-4) for 20 min. The coated fruits were air-dried and stored at ambient temperature (25 ± 2 °C and 50 ± 5%RH). Weight loss and visual appearance were monitored daily over a 15-day period to assess the preservation efficacy.

### 2.6. Statistical Analysis

All data presented with standard deviation were obtained from at least three independent replicates and are expressed as the mean ± SD.

## 3. Results and Discussion

### 3.1. Microstructure and Intermolecular Interactions of Composite Films

#### 3.1.1. Microstructural Morphology

**Figure 2:**
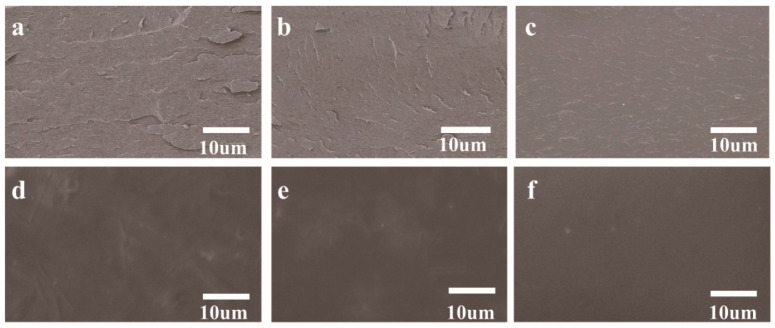
Microstructural morphology of the composite films. Cryo-fractured cross-sections of (**a**) Neat SPI, (**b**) SD-8, and (**c**) SDP-4 films. Surface morphology of (**d**) Neat SPI, (**e**) SD-8, and (**f**) SDP-4 films.

The microstructural evolution of the composite films, which provides crucial insights into the synergistic effects of DACNC and PA, was investigated by observing their cryo-fractured cross-sections and surfaces via SEM (Figure 2). The neat SPI film exhibited a rough, heterogeneous cross-section characterized by distinct lamellar strata and fissures (Figure 2a), a typical morphology indicative of a loosely aggregated protein network and inherent brittleness. Concurrently, its surface, while largely continuous, presented observable micropores and fine cracks (Figure 2d), which are common defects attributed to protein self-aggregation during the film drying process [19].

Upon the introduction of DACNC, the film’s internal architecture was markedly improved. The cross-section of the SD-8 film (Figure 2b) became significantly more compact and uniform, with a substantial reduction in the lamellar features. This structural enhancement is attributed to the formation of a robust covalent network via Schiff base reactions, which fortified the matrix’s continuity and cohesion. Correspondingly, the film surface (Figure 2e) appeared smoother and denser, confirming the effective filling of interstitial voids by the cross-linked network. This outcome demonstrates a clear superiority over the mere physical reinforcement offered by unmodified CNCs, where nanoparticle aggregation can still compromise matrix integrity [7].

A profound optimization of the microstructure was achieved with the further incorporation of PA. The cross-section of the SDP-4 film (Figure 2c) presented a dense, homogeneous, and vitreous-like fracture surface, entirely devoid of discernible defects. This morphology provides direct evidence of a potent synergistic reinforcement, wherein the extensive hydrogen-bonding network mediated by PA effectively permeates and consolidates the DACNC-established covalent framework. The corresponding surface (Figure 2f) was impeccably smooth and featureless, confirming the excellent miscibility and ordered molecular arrangement within the ternary system. This highly integrated and uniform microstructure is superior to that of films modified with small-molecule polyphenols, which often induce phase separation at higher concentrations [20]. Collectively, the SEM results confirm that the strategic combination of DACNC-mediated covalent cross-linking and PA-mediated non-covalent networking successfully constructs a highly compact and stable multi-network architecture. Although higher magnification observation may reveal finer details, existing images clearly demonstrate that the cracks and layered structures visible in pure SPI films (Figure 2a) have been eliminated. This provides direct evidence that the matrix has become more complete and cohesive, laying the structural foundation for the enhancement of the film’s macroscopic properties.

#### 3.1.2. Structural Characterization

**Figure 3:**
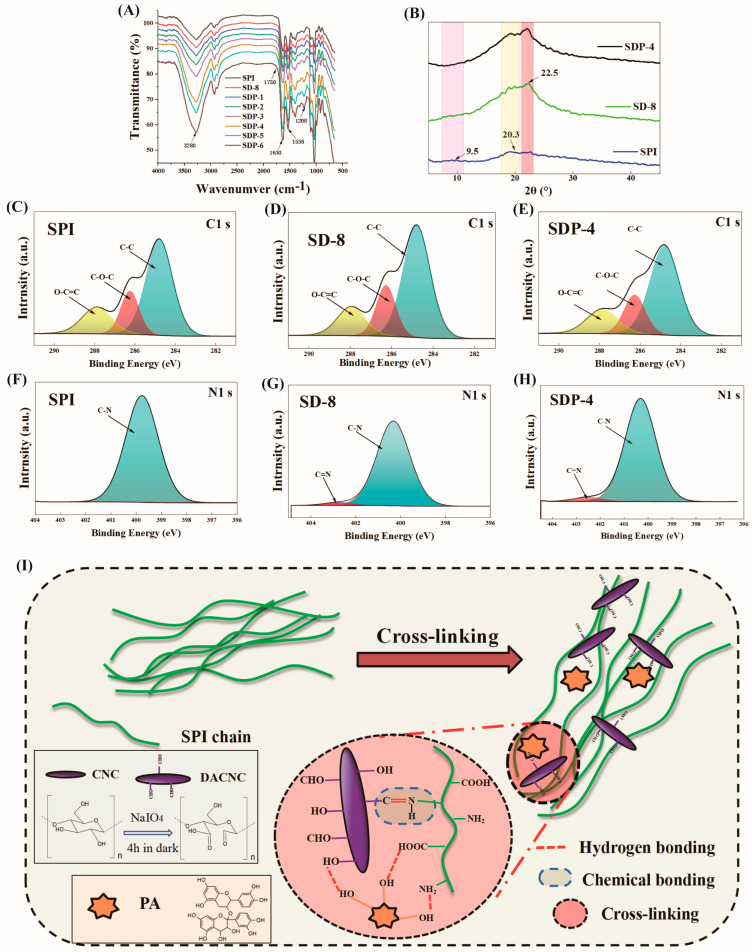
Structural characterization of the composite films. (**A**) FTIR spectra, and (**B**) XRD patterns of SPI, SD-8, and SDP-4 films. High-resolution XPS C1s spectra of (**C**) SPI, (**D**) SD-8, and (**E**) SDP-4 films. High-resolution XPS N1s spectra of (**F**) SPI, (**G**) SD-8, and (**H**) SDP-4 films. (**I**) Schematic illustration of the formation mechanism for the synergistic multi-network in the SDP composite film.

The formation mechanism of the hierarchical multi-network structure was further elucidated at the molecular level through a combination of spectroscopic and diffraction techniques (Figure 3).

FTIR Spectroscopy was employed to identify the key intermolecular interactions (Figure 3A). The neat SPI film displayed characteristic protein absorption peaks at 1652 cm^−1^ (Amide I, primarily C=O stretching) and 1538 cm^−1^ (Amide II, N-H bending and C-N stretching). Upon the introduction of DACNC (SD-8 film), a new shoulder peak emerged around 1730 cm^−1^, attributable to the C=O stretching of residual aldehyde groups on DACNC. More importantly, a distinct new absorption peak appeared at approximately 1630 cm^−1^, which is assigned to the C=N stretching vibration of the imine group formed via the Schiff base reaction between the aldehyde groups of DACNC and the primary amino groups of SPI. This peak partially overlaps with the Amide I band, causing it to broaden and shift, providing direct evidence for the formation of a covalent cross-linking network [21]. The systematic changes upon the addition of PA (SDP series) confirmed the establishment of the secondary, non-covalent network. As PA concentration increased, the broad absorption band centered at ~3280 cm^−1^ (representing overlapping O-H and N-H stretching vibrations) progressively widened and intensified. This is a classic indicator of extensive hydrogen bond formation between the abundant phenolic hydroxyl groups of PA and the polar groups (e.g., -COOH, -NH_2_, -OH) on both SPI and DACNC chains [22]. Concurrently, characteristic peaks of the PA aromatic skeleton (~1605 cm^−1^) and phenolic C-O stretching (~1208 cm^−1^) became more pronounced, verifying the successful incorporation of PA into the matrix.

XRD was used to analyze the changes in the aggregated state of the polymer chains (Figure 3B). The neat SPI film exhibited two broad, amorphous halos centered at 2θ ≈ 9.5° and 20.3°, corresponding to the α-helix and β-sheet secondary structures of the protein, respectively. The introduction of DACNC (SD-8 film) resulted in the appearance of a new, relatively sharp diffraction peak at 2θ ≈ 22.5°. which corresponds to the characteristic reflection of the Cellulose I crystalline structure (specifically attributed to the (002) plane), confirming the inherent crystallinity of the DACNC. The retention of this crystalline core within the DACNC, despite the preparation process, ensures it functions as a rigid reinforcement element integrated into the SPI matrix [15]. Interestingly, after the synergistic modification with both DACNC and PA (SDP-4 film), the original protein diffraction peaks became less defined, while the cellulose peak at 22.5° remained. This suggests that the formation of the dense, dual cross-linked network disrupted the inherent, ordered packing of the SPI molecular chains, leading to a more amorphous protein phase. This structural disruption is a positive indicator that strong intermolecular interactions have effectively reorganized the matrix at a supramolecular level.

The broad halo of the SPI film (at ca. 20°) primarily represents the amorphous structure of the protein. The reduction in the half-width of this halo upon DACNC incorporation indicates an increase in the structural order of the composite film. This is not due to an expansion of crystalline regions but is attributed to the combined effect of the rigid covalent backbone formed by DACNC and the multi-point hydrogen-bonding filling network formed by PA, which restricts the random movement of protein chains, inducing local molecular compaction and quasi-ordering and leading to a transition from a loose amorphous state to a densely packed quasi-ordered aggregate state.

The characteristic crystalline diffraction peaks of DACNC (e.g., 2θ ≈ 22.5°) were masked and diluted by the strong, broad amorphous halo of the SPI matrix and attenuated by the chemical cross-linking. This is due to the low content of DACNC (8 wt%) and the partial disruption of its surface structure during oxidation, which are key factors influencing the XRD features of nanofillers uniformly dispersed and covalently cross-linked within a highly amorphous polymer matrix.

It is noted that quantitative peak deconvolution was not performed on the diffractograms. This methodological choice was made because the composite system features a highly amorphous SPI matrix, resulting in severe overlapping between the broad amorphous hump and the relatively weak DACNC characteristic peaks. In such complex systems, the results of quantitative deconvolution are often highly susceptible to systematic errors and uncertainty. Consequently, we utilized the XRD data primarily for qualitative analysis, focusing on the preservation of the DACNC crystalline core and the clear shift in the main amorphous peak (indicating matrix densification and restricted chain mobility), which already provides robust evidence for the proposed hierarchical multi-network structure.

XPS provided further chemical evidence from the film surface. The high-resolution C1s spectrum of the neat SPI film (Figure 3C) was deconvoluted into three main components: C-C/C-H (~284.8 eV), C-O/C-N (~286.2 eV), and O-C=O (~288.0 eV). For the SD-8 film (Figure 3D), the relative intensity of the C-O/C-N peak increased, consistent with the introduction of the oxygen-rich DACNC. Crucially, in the N1s spectrum of the SD-8 film (Figure 3G), a new component appeared at a higher binding energy of ~401.5 eV, which can be assigned to the nitrogen atom in the newly formed C=N imine bond, providing unequivocal evidence for the Schiff base reaction [23]. For the SDP-4 film (Figure 3E), the C1s spectrum showed a significant increase in the C-C component, reflecting the high proportion of aromatic rings in the incorporated PA. The N1s spectrum of the SDP-4 film (Figure 3H) retained the C=N signal, confirming the stability of the covalent network. Collectively, the multi-technique analyses confirm the successful construction of a hierarchical dual-network system, where DACNC forms a covalent backbone via Schiff base reactions, and PA integrates into this framework through extensive hydrogen bonding, fundamentally altering the film’s chemical and physical structure.

The underlying mechanism of this synergistic reinforcement is schematically illustrated in Figure 3I. Initially, the SPI chains form a weak, physically entangled network. The introduction of DACNC, synthesized via selective oxidation of CNC, initiates the primary cross-linking event. The abundant aldehyde groups (-CHO) on the DACNC surface react with the primary amino groups (-NH_2_) on the SPI chains (e.g., from lysine residues) to form robust C=N imine bonds, creating a stable, covalently cross-linked backbone (Chemical bonding). Subsequently, PA, with their numerous phenolic hydroxyl (-OH) groups, are introduced. These PA molecules act as a “molecular glue,” establishing an extensive, secondary network of multi-point hydrogen bonds with the polar groups (e.g., -OH, -COOH, -NH_2_) present on both the SPI chains and the DACNC surfaces (Hydrogen bonding). This dual-network architecture, composed of a rigid covalent skeleton interwoven with a dense non-covalent matrix, effectively transforms the loose protein assembly into a highly compact, integrated, and stable composite structure.

### 3.2. Mechanical and Thermal Properties

The macroscopic performance of the composite films was systematically evaluated to establish the structure property relationships, with mechanical integrity and thermal stability being the primary indicators of their structural robustness.

**Figure 4:**
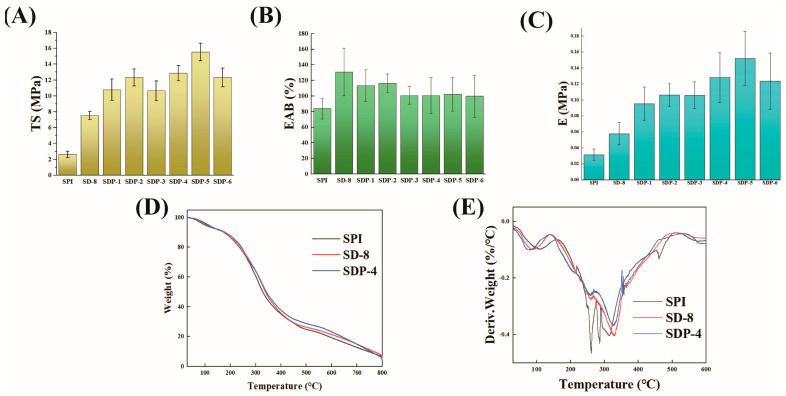
Mechanical and thermal properties of the composite films. (**A**) Tensile strength (TS), (**B**) Elongation at break (EAB), and (**C**) Young’s Modulus of all composite films. (**D**) TGA and (**E**) DTG curves of SPI, SD-8, and SDP-4 films.

The mechanical properties, crucial for the film’s durability in packaging applications, were quantified by TS and EAB (Figure 4A,B, and Table 2). The neat SPI film, constrained by its weak non-covalent network, exhibited poor mechanical performance with a low TS of only 2.63 MPa and an EAB of 83.9%. The sole introduction of DACNC (SD-8 film) induced a substantial reinforcement, increasing the TS by 186% to 7.52 MPa. This enhancement is a direct consequence of the robust covalent cross-linking network, which effectively transfers stress throughout the matrix and is superior to the reinforcement typically achieved by the simple physical incorporation of unmodified CNC [24]. The Young’s Modulus, a measure of film stiffness, followed a similar trend to tensile strength, increasing from 0.03 MPa for the neat SPI film to 0.15 MPa for the SDP-5 film. This confirms that the multi-network structure not only strengthens the film but also significantly increases its rigidity.

Remarkably, a profound synergistic effect was observed upon the further incorporation of PA. The TS of the composite films initially increased with PA content, reaching an impressive peak of 15.54 MPa for the SDP-5 film. This represents a 491% and 107% improvement over the neat SPI and SD-8 films, respectively, providing compelling evidence for the synergistic reinforcement mechanism. This high level of tensile strength significantly surpasses that reported for SPI films modified with most single components, such as small-molecule polyphenols or other polysaccharides [25]. The synergy arises from the hierarchical multi-network architecture, where the DACNC covalent network provides a rigid skeleton while the pervasive PA hydrogen-bonding network acts as a molecular “mortar,” filling voids and enhancing interfacial adhesion, which effectively dissipates stress and prevents crack propagation. However, excessive PA content (SDP-6) led to a slight decrease in TS, likely due to the self-aggregation of PA molecules, which can introduce defects and disrupt network uniformity.

The EAB values showed that the introduction of DACNC initially increased the film’s flexibility (130.7% for SD-8), but the subsequent addition of PA resulted in a gradual decrease in EAB, stabilizing at around 100–110%. The observed mechanical performance is a direct outcome of the synergistic dual-network strategy. Specifically, the substantial enhancement in Young’s modulus is principally attributed to the formation of the rigid covalent backbone via Schiff base linkages between SPI and DACNC, which provides high stiffness and resistance to elastic deformation. Crucially, the relative stability of the elongation at break indicates that the film maintains a desirable level of toughness. This stable EAB is maintained by the flexible hydrogen-bonding filling network provided by PA. This dense PA network effectively disperses localized stress and restricts crack propagation, allowing the composite to withstand substantial plastic deformation before failure, thereby realizing an excellent balance between high strength and adequate toughness.

TGA was conducted to evaluate the thermal stability of the films (Figure 4D,E). All samples exhibited an initial weight loss below 150 °C, attributed to the evaporation of bound water and glycerol. The main thermal degradation event for the neat SPI film, corresponding to the cleavage of peptide bonds and protein side chains, occurred at a maximum decomposition temperature (Tmax) of approximately 265 °C, as determined from the DTG curve. A significant enhancement in thermal stability was observed upon modification. For the SD-8 film, the Tmax shifted dramatically to 320 °C. This substantial increase is a direct result of the covalent network, which restricts the thermal motion of protein chains and elevates the energy required for their decomposition. The thermal stability was further improved in the SDP-4 film, with its Tmax increasing to 330 °C. This demonstrates the dual contribution of PA: (1) its intrinsic high thermal stability stemming from its aromatic structure, and (2) its ability to form a denser, synergistic char layer with DACNC at high temperatures, which acts as a thermal and mass transfer barrier, delaying the thermo-oxidative degradation of the protein matrix. These TGA results unequivocally confirm that the synergistic multi-network structure imparts superior thermal stability to the composite film, a crucial attribute for packaging applications.

While the TGA curves exhibit a generally similar profile—reflecting that the low content of cross-linkers does not fundamentally alter the principal thermal degradation pathway of the bulk SPI—the structural modification is quantitatively confirmed by the significant increase in the maximum degradation temperature (T_max_). The T_max_ shifts substantially from 265 °C (for pure SPI film) to 330 °C (for SDP-4). This pronounced 65 °C enhancement serves as direct evidence that the formation of the rigid multi-network structure successfully restricts the segmental motion of the polymer chains, thus elevating the energy barrier required for thermal decomposition and confirming the enhanced thermal stability predicted by the increased modulus. Conversely, the dramatic enhancement in mechanical properties (e.g., 491% increase in TS for SDP-4) is not governed by covalent bond stability at high temperature but by the efficiency of the supramolecular network structure at ambient conditions. This enhancement is achieved through significantly increased cross-link density and efficient stress dispersion by the multi-network structure. Thus, the TGA results confirm the inherent thermal stability is maintained, while the mechanical results validate the success of the physical optimization and defect-filling strategy, confirming that the two findings are consistent and complementary.

### 3.3. Optical Properties and UV-Shielding Capability

**Figure 5:**
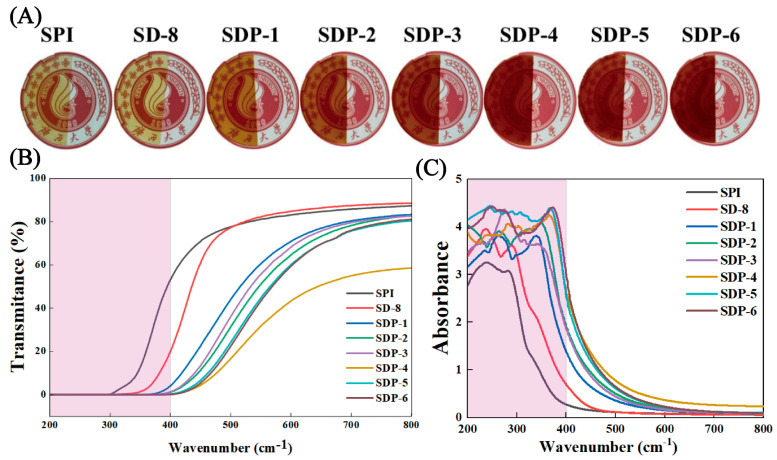
Optical properties of the composite films. (**A**) Digital photographs of the films placed over a university logo. (**B**) UV-vis light transmittance spectra and (**C**) UV-vis light absorbance spectra of the films.

The optical properties of packaging films, including visual appearance, color, and light transmittance, are crucial as they directly influence consumer acceptance and the protection of light-sensitive food products. As illustrated in Figure 5A, all films exhibited a smooth and homogeneous appearance. The neat SPI film was transparent with a light-yellowish hue. With the increasing incorporation of PA, the films progressively darkened, transitioning to a reddish-brown color characteristic of proanthocyanidins. Despite this deepening in color, all films maintained good transparency, allowing the underlying university logo to remain clearly visible, which is a desirable attribute for food packaging.

This visual observation was quantified by colorimetric analysis (Table 3). The neat SPI film exhibited the highest L* value (lightness) of 80.70. Upon the addition of PA, the L* values of the SDP series films systematically decreased, reaching a minimum of 39.39 for SDP-6, confirming the darkening effect. Concurrently, the a* (redness) and b* (yellowness) values significantly increased with PA concentration, consistent with the observed color shift. This trend unequivocally demonstrates that the film’s color is directly correlated with the PA content, a factor that can be tailored for specific applications, such as protecting foods from visible light.

Beyond visual appearance, the UV-light barrier performance of the films was evaluated (Figure 5B,C), revealing one of the most significant functional improvements. The neat SPI and SD-8 films offered minimal protection, showing high transmittance across the UV spectrum (200–400 nm). In stark contrast, all PA-containing films (SDP series) demonstrated outstanding UV-shielding capabilities. The light transmittance in the entire UVA, UVB, and UVC regions (200–400 nm) was reduced to virtually zero. This near-complete blockage of harmful UV radiation is attributed to the abundant aromatic rings and conjugated π-electron systems within the PA molecular structure, which effectively absorb high-energy photons through π-π* transitions [17]. This level of UV protection is superior to that of many films modified with other natural extracts and is comparable to systems incorporating synthetic UV absorbers or specialized nanoparticles [26,27]. This excellent UV-shielding performance is highly desirable for active food packaging, as it can effectively prevent the photo-oxidation of lipids, vitamins, and pigments in food, thereby significantly extending shelf life.

### 3.4. Water Responsive Properties

The interaction with water, encompassing both bulk water resistance and moisture barrier properties, is a paramount performance criterion for SPI-based films in food packaging. The film’s response to water in both vapor and liquid states was systematically evaluated.

**Figure 6:**
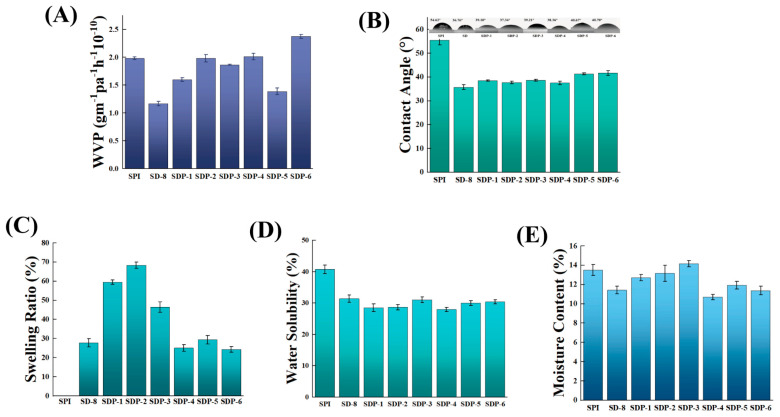
Water responsive properties of the composite films. (**A**) Water vapor permeability (WVP), (**B**) Water contact angle (WCA), (**C**) Swelling Ratio (*SR*), (**D**) Water solubility (*WS*), and (**E**) Moisture content (*MC*) of the films.

A critical indicator of moisture barrier performance, the water vapor permeability (WVP), showed significant improvement upon modification (Figure 6A). The neat SPI film exhibited a high WVP of 2.0 × 10^−10^ g·m^−1^·h^−1^·Pa^−1^. In contrast, the WVP of the modified films was substantially reduced, reaching a minimum of 1.2 × 10^−10^ g·m^−1^·h^−1^·Pa^−1^ for the SD-8 film, representing a 40% reduction. This enhancement is a direct consequence of the dense, multi-network structure confirmed by SEM. The covalently cross-linked DACNC network, reinforced by the PA-mediated hydrogen bonds, creates a highly tortuous path for water molecules, effectively impeding their diffusion through the film matrix. This level of barrier enhancement is a notable advantage of the synergistic network strategy compared to simple polymer blending [28]. Interestingly, the addition of PA led to a slight increase in WVP compared to the SD-8 film, which can be attributed to the hydrophilic nature of PA itself.

The film’s structural integrity in the presence of liquid water was further confirmed by water solubility and *SR* tests (Figure 6C,D). The neat SPI film displayed a high *WS* of 40.7% and completely disintegrated upon prolonged immersion, making *SR* measurement impossible. The introduction of DACNC (SD-8 film) dramatically reduced the *WS* to 31.3%, demonstrating the effectiveness of covalent cross-linking in locking the protein chains and preventing their dissolution. With the addition of PA, the *WS* of the SDP films was further reduced, reaching a minimum of 27.8% for the SDP-3 film. This remarkable improvement in water stability is attributed to the synergistic effect of the dual network, which is more effective at resisting water penetration and dissolution than networks formed by single cross-linkers or non-covalent interactions alone [29]. The *SR* values showed a trend where higher PA content (SDP-3 to SDP-6) generally reduced swelling by forming an even denser hydrogen-bond network that restricted water ingress.

The WCA measurement provides insight into the surface wettability of the films (Figure 6B). The neat SPI film exhibited a WCA of 54.6°, characteristic of a hydrophilic surface. Intriguingly, the incorporation of both DACNC and PA led to a decrease in WCA to approximately 36–42°. This indicates an increase in surface hydrophilicity, which can be attributed to the introduction of additional free hydroxyl groups from the DACNC and, particularly, the PA molecules exposed on the film surface. It is crucial to distinguish this surface phenomenon from the bulk properties. While the surface became more wettable, the aforementioned improvements in WVP, *WS*, and *SR* unequivocally demonstrate that the film’s overall water resistance is substantially enhanced. This confirms that the compactness of the internal network structure, rather than surface chemistry, is the dominant factor governing the material’s barrier performance and stability against water. The moisture content of the films (Figure 6E) showed a complex trend, reflecting the delicate balance between the introduction of hydrophilic groups and the formation of a compact, water-restricting network.

It is noteworthy that the incorporation of DACNC and PA resulted in a smoother surface morphology (Figure 2) but a more hydrophilic surface (lower WCA, Figure 6B). This observation can be understood by considering the interplay between surface topography and surface chemistry. According to the Wenzel model, increasing the roughness of an intrinsically hydrophilic surface typically enhances its hydrophilicity (i.e., decreases WCA). Our results, which show the opposite trend with respect to topography, indicate that the change in surface chemistry is the dominant factor. The incorporation of proanthocyanidins introduces a high density of polar phenolic hydroxyl groups onto the film surface, significantly increasing its intrinsic hydrophilicity. This chemical effect evidently overrides the physical effect of reduced surface roughness, leading to the observed decrease in water contact angle.

The mechanical performance exhibits a clear correlation with the water stability: the significant increase in E is logically consistent with the simultaneous substantial reduction in both swelling ratio and water solubility (Figure 4C and Figure 6C,D). This strong correlation confirms that these properties are all governed by the same structural factor: network densification. The dual network operates as follows: the DACNC covalent linkages provide chemical resistance to dissolution (low *WS*) and enhance stiffness (high E); simultaneously, the PA-induced filling network dramatically reduces the available free volume within the matrix, thereby physically obstructing water molecule penetration and uptake (low *SR*). Furthermore, the relatively stable moisture content results from a counterbalancing mechanism. Although PA possesses hydrophilic hydroxyl groups that tend to absorb moisture, this tendency is largely offset by the overall reduction in internal porosity and accessible free volume due to the profound network densification. This explains why the film achieves superior water resistance (low *SR*) without a drastic loss of equilibrium moisture content.

### 3.5. Antioxidant and Antimicrobial Activities

#### 3.5.1. Antioxidant Activity

The ultimate goal of developing the multi-network composite film is to create an active packaging material with potent biological functions. Therefore, the film’s antioxidant and antimicrobial activities, along with its practical efficacy in food preservation, were systematically investigated.

**Figure 7:**
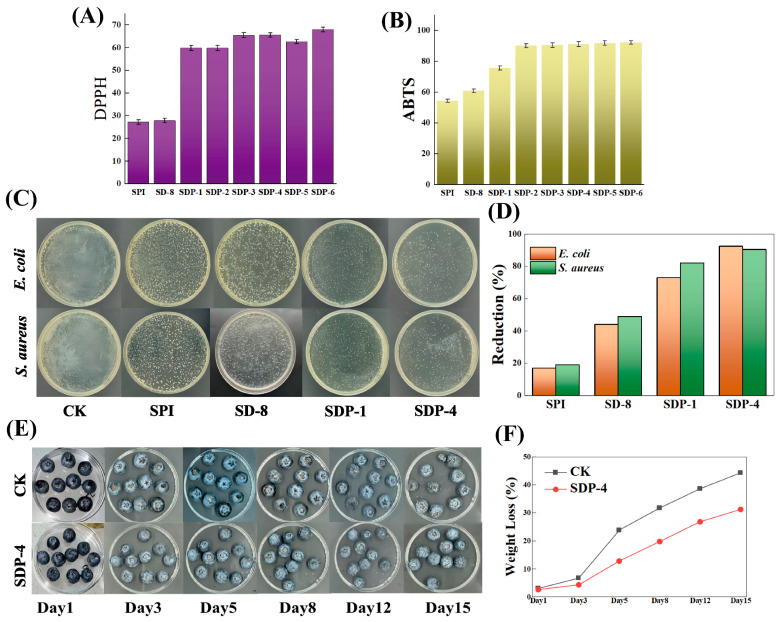
Bioactivity and preservation application of the composite films. (**A**) DPPH and (**B**) ABTS radical scavenging activities. (**C**) Digital photographs of bacterial colonies (*E. coli* and *S. aureus*) after treatment with different film solutions and (**D**) corresponding bacterial reduction rates. (**E**) Digital photographs showing the appearance of uncoated (CK) and SDP-4 coated blueberries during 15 days of storage. (**F**) Weight loss curves of the blueberries during storage.

The antioxidant capacity is a critical attribute for inhibiting the oxidative deterioration of food. This was evaluated via DPPH and ABTS radical scavenging assays (Figure 7A,B). The neat SPI and SD-8 films exhibited negligible antioxidant activity, confirming that neither the protein matrix nor the DACNC cross-linker possesses significant radical scavenging capabilities. In stark contrast, the incorporation of PA endowed the SDP films with potent antioxidant properties in a concentration-dependent manner. As the PA content increased, the scavenging rates for both DPPH and ABTS radicals rose dramatically. The SDP-6 film demonstrated the highest capacity, with scavenging rates of 67.8% for DPPH and 93.2% for ABTS radicals. This pronounced enhancement unequivocally confirms that PA is the primary contributor to the film’s antioxidant function, owing to the abundant phenolic hydroxyl groups in its structure that act as hydrogen donors to efficiently neutralize free radicals [29,30,31].

#### 3.5.2. Antimicrobial Activity

Effective inhibition of microbial growth is fundamental for ensuring food safety. The antibacterial performance against *S. aureus* (Gram-positive) and *E. coli* (Gram-negative) was therefore assessed (Figure 7C,D). The control (CK) and neat SPI plates showed prolific bacterial growth. The SD-8 film exhibited a moderate level of inhibition, with reduction rates of 45.3% and 49.5% against *E. coli* and *S. aureus*, respectively. This can be attributed to the altered surface properties and improved structural integrity from DACNC cross-linking. A qualitative leap in antimicrobial performance was achieved with the introduction of PA. The SDP films demonstrated exceptionally strong and broad-spectrum activity, culminating in the SDP-4 film, which achieved high reduction rates of 95.6% against *S. aureus* and 96.2% against *E. coli*. This potent bactericidal effect is primarily due to PA’s ability to disrupt the integrity of bacterial cell membranes, inhibit essential enzyme activities, and interfere with cellular metabolism [14,17,32].

Finally, a blueberry preservation experiment was conducted to validate the practical efficacy of the optimized film (Figure 7E,F). The visual changes over 15 days clearly demonstrated the superior protective effect of the SDP-4 coating. By day 12, the uncoated control (CK) group exhibited severe fungal growth (mold) and tissue collapse. In stark contrast, the SDP-4 coated blueberries remained largely intact, showing only minor wrinkling but no visible microbial spoilage. The weight loss data quantitatively corroborated these observations (Figure 7F). The control group experienced rapid weight loss, reaching 47.1% by day 15. Conversely, the weight loss of the SDP-4 group was significantly retarded, reaching only 33.5%. This dual-action protection is attributed to: (1) the dense multi-network structure forming an effective physical barrier against moisture evaporation, and (2) the potent antioxidant and antimicrobial activities inhibiting the metabolic activities of both spoilage microorganisms and the fruit itself. This experiment confirms that the synergistic design of the SDP film successfully translates its excellent physicochemical properties and bioactivities into a tangible extension of shelf life for perishable fruits [33,34]. The reduced weight loss is attributed to the film’s enhanced water vapor barrier properties (Figure 6A), which creates a modified atmosphere around the fruit, slowing respiration and transpiration. The potent antioxidant activity of the PA-containing film likely scavenges reactive oxygen species generated during senescence, which would be expected to preserve endogenous antioxidants like anthocyanins and other phenolic compounds, thereby maintaining the fruit’s nutritional quality and color. Similarly, the strong antimicrobial properties directly inhibit the growth of spoilage fungi, as visually confirmed in Figure 7E.

## 4. Limitations and Future Perspectives

The blueberry preservation study, while demonstrating clear efficacy in extending shelf life, was limited to macroscopic evaluation (visual appearance and weight loss). Future studies should incorporate quantitative biochemical analyses, such as measuring changes in total phenolic content, anthocyanin concentration, firmness, and soluble solids, to provide a more comprehensive understanding of the preservation mechanism.

A crucial aspect for any material intended for food packaging is a rigorous toxicological safety assessment. While all components used in this study are derived from natural and biodegradable sources, we emphasize that natural origin is not a substitute for empirical safety validation. A major limitation of the current work is the lack of in vitro cytotoxicity testing. Future work must therefore include a comprehensive biocompatibility assessment of the composite films. This would involve quantitative assays, such as the MTT assay using relevant cell lines (e.g., Caco-2 intestinal cells), to evaluate the potential for leachates from the film to induce cytotoxic effects. Such studies are a necessary prerequisite for establishing the safety of the films for direct food contact and for advancing this material system towards practical application.

Furthermore, although the components are biodegradable, the influence of cross-linking on the composite film’s degradation rate remains to be quantified. Future work should include standard soil burial or controlled composting experiments to systematically assess the film’s degradation profile and ensure its end-of-life environmental compatibility.

## 5. Conclusions

In conclusion, the hierarchical multi-network approach successfully transformed SPI into a high-performance, functional biocomposite. Crucially, the overall investigation demonstrates a clear and definitive correlation between the structural foundation and the resulting macroscopic properties. The structural evidence (FTIR, XPS, XRD) confirms the successful formation of the dual network, where the covalent linkages provide the core rigidity and thermal resistance, and the PA-induced densification dictates the mechanical balance, water stability, and enhanced functional attributes.

This study successfully demonstrates that a synergistic multi-network strategy, combining covalent cross-linking by DACNC with non-covalent reinforcement by PA, can effectively transform brittle SPI into a high-performance, bioactive material. The resulting composite film, underpinned by a dense and stable architecture, exhibited a remarkable 491% increase in tensile strength to 15.54 MPa, alongside a significant elevation in thermal stability to 330 °C. Crucially, this structural enhancement was coupled with potent functionalities, including complete UV-light blockage, high radical scavenging (93.2% for ABTS), and strong broad-spectrum antimicrobial activities (up to 96.2% inhibition). The practical efficacy of these integrated properties was unequivocally validated by the significant extension of fresh blueberry shelf life. Ultimately, this work validates that the rational design of synergistic multi-networks is a powerful framework for developing advanced bio-based materials with tailored functionalities, holding great promise for sustainable applications in food packaging and beyond.

## Figures and Tables

**Table 1:** Composition of the SDP composite films.

Sample	SPI (g)	Glycerol (g)	DACNC (g)	PA (g)
SPI	0.6	0.18	0	0
SD-8	0.6	0.18	0.048	0
SDP-1	0.6	0.18	0.048	0.006 (1%)
SDP-2	0.6	0.18	0.048	0.012 (2%)
SDP-3	0.6	0.18	0.048	0.018 (3%)
SDP-4	0.6	0.18	0.048	0.024 (4%)
SDP-5	0.6	0.18	0.048	0.030 (5%)
SDP-6	0.6	0.18	0.048	0.036 (6%)

The percentage (%) indicates the mass fraction of PA relative to SPI.

**Table 2:** Thickness and mechanical properties of SPI, SD-8, and SDP composite films.

Sample	Thickness (mm)	TS (MPa)	EAB (%)	E (MPa)
SPI	0.116 ± 0.018	2.63 ± 0.40	83.9 ± 13.2	0.0314
SD-8	0.117 ± 0.023	7.55 ± 0.53	130.8 ± 30.4	0.0577
SDP-1	0.113 ± 0.016	10.79 ± 1.35	113.3 ± 20.4	0.0952
SDP-2	0.119 ± 0.013	12.35 ± 1.06	116.4 ± 11.9	0.1061
SDP-3	0.125 ± 0.011	10.68 ± 1.23	101.0 ± 11.2	0.1058
SDP-4	0.143 ± 0.039	12.88 ± 0.95	100.6 ± 23.4	0.1280
SDP-5	0.155 ± 0.026	15.54 ± 1.11	102.4 ± 21.6	0.1518
SDP-6	0.149 ± 0.025	12.33 ± 1.18	99.9 ± 26.9	0.1235

**Table 3:** Color parameters (L*, a*, b*, and ΔE) of the composite films.

Sample	L*	a*	b*	ΔE
SPI	80.70 ± 1.19	6.52 ± 0.89	35.80 ± 2.37	3.96 ± 0.46
SD-8	84.49 ± 0.37	2.12 ± 0.12	31.81 ± 1.45	2.4 ± 0.41
SDP-1	63.42 ± 0.89	18.40 ± 0.68	47.67 ± 1.48	1.97 ± 0.20
SDP-2	55.84 ± 1.39	25.07 ± 1.87	50.69 ± 0.80	2.91 ± 1.67
SDP-3	48.36 ± 1.19	28.87 ± 0.43	54.21 ± 0.80	2.11 ± 0.29
SDP-4	39.71 ± 1.11	33.24 ± 3.29	52.66 ± 2.8	6.72 ± 1.06
SDP-5	40.95 ± 1.58	30.18 ± 0.65	53.15 ± 1.80	6.01 ± 1.69
SDP-6	39.39 ± 2.1	30.62 ± 0.69	48.84 ± 3.3	3.84 ± 0.87

## Data Availability

The raw data supporting the conclusions of this article will be made available by the authors on request.
